# Increased Synovial *CD14* mRNA Expression and Proportion of CD14^high^ Subsets in Early-Stage Hip Osteoarthritis: Propensity Matched Score Analysis

**DOI:** 10.3390/ijms232113622

**Published:** 2022-11-07

**Authors:** Yoshihisa Ohashi, Kentaro Uchida, Kensuke Fukushima, Masashi Satoh, Tomohisa Koyama, Maho Tsuchiya, Hiroki Saito, Katsufumi Uchiyama, Naonobu Takahira, Gen Inoue, Masashi Takaso

**Affiliations:** 1 Department of Orthopaedic Surgery, Kitasato University School of Medicine, 1-15-1 Minami-ku Kitasato, Sagamihara City 252-0374, Japan; 2 Department of Immunology, Kitasato University School of Medicine, 1-15-1 Minami-ku Kitasato, Sagamihara City 252-0374, Japan; 3 Department of Rehabilitation, Kitasato University School of Allied Health Sciences, 1-15-1 Minami-ku Kitasato, Sagamihara City 252-0375, Japan

**Keywords:** hip osteoarthritis, early-stage osteoarthritis, hip pain, CD14+ cells, CD14^high^ subsets

## Abstract

The pathophysiology of early-stage hip osteoarthritis (EOA) is not fully understood. Although a previous study in an age-unmatched cohort reported that the number of macrophages was increased in knee EOA compared to late OA (LOA), it remained unclear whether increased macrophages in EOA accurately reflect EOA pathology. We investigated the differences in *CD14* expression levels between EOA and LOA using age-unmatched and -matched cohorts. Synovial tissues were obtained from 34 EOA (Tönnis grades 0 and 1) and 80 LOA (Tönnis grades 2 and 3) patients. To correct for differences in demographics between patients with LOA and EOA, we also created propensity score-matched cohorts (16 EOA and 16 LOA). *CD14* expression and its association with pain was estimated in LOA and EOA before and after propensity matching. We performed flow cytometry on tissues from the 16 patients, with 8 from each group, to assess for CD14+ subsets in the cells. The *CD14* expression in EOA was higher than that in LOA both before and after propensity matching. The proportion of CD14^high^ subsets in EOA was higher than that in LOA. The *CD14* expression was associated with pain in EOA before matching. However, no difference was observed between the pain and *CD14* expression after matching in EOA. The increased *CD14* expression and the proportion of CD14^high^ subsets may be important features associated with hip EOA pathology. To accurately compare early and late OA, the analysis of a propensity score-matched cohort is necessary.

## 1. Introduction

Hip osteoarthritis (OA) is a degenerative joint disease that causes progressive morphological changes over time, resulting in impaired gait and reduced quality of life. It has been shown in early knee OA that therapeutic interventions in preliminary stages, prior to the onset of irreversible and chronic joint damage, are quite effective [1,2]. In patients with painful early hip OA, arthroscopic surgeries (AS), including synovectomy, labral repair and reconstruction, and cam osteochondroplasty for femoroacetabular impingement (FAI) and/or developmental dysplasia of the hip (DDH), have become widely used therapeutic interventions [3,4,5]. Although differences in the pathophysiology of early and late hip and knee OA should be differentiated [6], data on early hip OA is lacking.

Synovial inflammation is one of the peripheral factors signaling the onset of pain and OA progression [7,8,9,10], and is observed in both early hip and knee OA [11,12,13]. Monocytes and macrophages contribute to pain in multiple tissues, including the synovium of OA, through the production of pronociceptive molecules [14,15,16,17,18,19]. Recent evidence suggests that the activation of macrophages in synovial tissue leads to the inflammation and subsequent degradation of cartilage with osteophyte formation [20]. CD14 is a component of the lipopolysaccharide receptor complex; the principal receptor is Toll-like receptor 4, which is expressed on the surface of monocytes and macrophages [21]. We previously reported that CD14+ cells expressed greater quantities of inflammatory cytokines than CD14− cells in synovium derived from late knee OA patients [22]. Daghestani et al. suggested that CD14 was positively associated with the progression of joint space narrowing, osteophyte formation, and pain intensity in synovial fluid from patients with knee OA, including all stages in the Kellgren and Lawrence (KL) classification [23]. A previous study reported that synovium in early knee OA had a higher proportion of macrophages compared to late knee OA [11], suggesting that macrophages play an important role in early OA. However, the early knee OA group was younger than the late OA group, and given that the proportion of macrophages can differ with aging [24], it remained unclear whether the increase in macrophages in early OA accurately reflected early OA pathology. We considered that the comparison of early and late hip OA (EOA, LOA) in cohorts matched with respect to likely confounding variables would reveal whether macrophages contribute to EOA pathology.

In this study, we investigated the differences in *CD14* expression levels between EOA and LOA using age-unmatched and -matched cohorts.

## 2. Results

### 2.1. Patients’ Clinical and Radiographic Data in EOA and LOA before and after Propensity Matching

Table 1 shows the comparisons of patient background factors, and clinical and radiographic evaluations in patients with EOA and LOA who underwent RT-PCR analysis before and after propensity matching. There were no significant differences in gender ratio, weight, body mass index, or pain intensity between early and late disease. Before propensity matching, patients with EOA were significantly younger and taller than those with LOA (*p* < 0.001). However, after matching, there were no statistically significant differences between EOA and LOA. In radiographic assessments, the proportion of patients with each Tönnis grade differed significantly between EOA and LOA (*p* < 0.001) both before and after matching. Before matching, there were 27 and 7 patients with Tönnis grades of 0 and 1 in EOA, respectively, and 21 and 59 patients with Tönnis grades of 2 and 3 in LOA, respectively. After matching, there were 11 and 5 patients with Tönnis grades of 0 and 1 in early hip OA, respectively, and 3 and 13 patients with Tönnis grades of 2 and 3 in LOA, respectively. Before matching, α-angles ≥ 60° or lateral center-edge (LCE) angles ≤ 25° were identified in 11 (32.4%) and 14 (41.2%) patients with EOA, respectively. Five patients had combined α-angles ≥ 60° and LCE angles ≤ 25°. After matching, α-angles ≥ 60° or lateral center-edge (LCE) angles ≤ 25° were identified in 2 (12.45%) and 10 (62.5%) patients with early hip OA, respectively. Two patients had combined α-angles ≥ 60° and LCE angles ≤ 25°.

### 2.2. Differences in Synovial CD14 Expression in Patients with Early and Late Osteoarthritis

**Figure 1:**
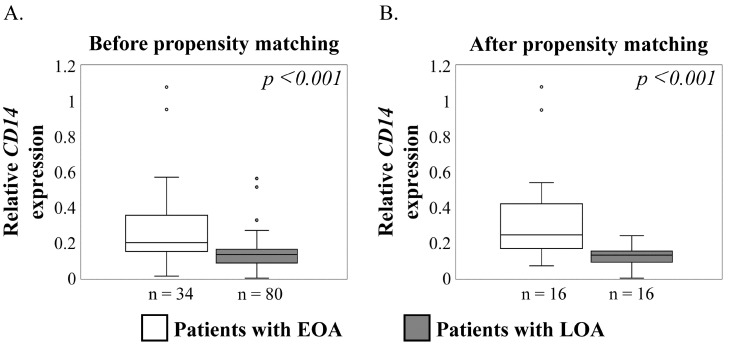
Comparison of synovial *CD14* expression in patients with early and late hip osteoarthritis (EOA and LOA, respectively). Synovial *CD14* expression in the before (**A**) and after (**B**) propensity matching cohorts. *X* axis: white and gray boxes represent the patients with EOA and LOA, respectively. *Y* axis: relative *CD14* mRNA expression. The Mann–Whitney *U*-test was used for analysis. *p* < 0.05 indicates statistical significance. Boxes represent median and IQR (interquartile range, 25–75th percentiles); small circle represent values outside 1.5 × IQR.

Figure 1 identifies the different expressions of *CD14* in synovial cells from patients with EOA and LOA before and after propensity matching. *CD14* expression was significantly higher in EOA than in LOA before (Figure 1A, *p* = 0.001) and after matching (Figure 1B, *p* < 0.001).

### 2.3. Differences in Synovial CD14 Expression and Pain Intensity

**Figure 2:**
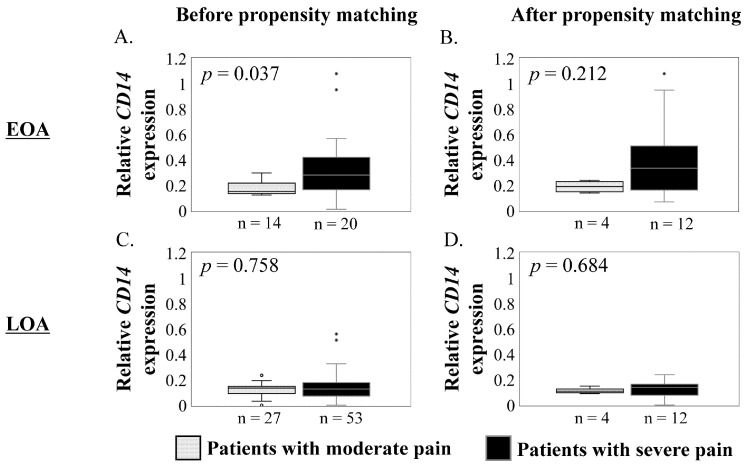
Comparisons of synovial *CD14* expression between moderate and severe pain in patients with early hip osteoarthritis (EOA) and late hip osteoarthritis (LOA). Synovial *CD14* expression (*y* axis) in EOA patients with moderate (gray boxes) and severe (black boxes) pain before (**A**) and after (**B**) propensity matching. Synovial *CD14* expression in LOA patients with moderate and severe pain before (**C**) and after (**D**) propensity matching. Boxes represent median and IQR (interquartile range, 25–75th percentiles); whiskers represent range within 1.5 × IQR; and small circle represent values outside 1.5 × IQR. *Y* axis, relative *CD14* mRNA expression. The Mann–Whitney *U*-test was used for analysis. *p* < 0.05 indicates statistical significance.

We investigated the differences in synovial *CD14* expression in severe or moderate pain (visual analog scale [VAS] pain ≥ 5 or < 5) in patients with EOA and LOA. In EOA, the *CD14* expression in synovium was significantly higher in cases with severe pain than with moderate pain before propensity matching (*p* = 0.037, Figure 2A). However, there was no significant difference after propensity matching (*p* = 0.212, Figure 2B). In LOA, there were no significant differences in *CD14* expression between the severe and moderate pain before and after propensity matching (*p* = 0.758, and *p* = 0.684, respectively; Figure 2C,D).

### 2.4. Differences in the Proportion of CD14+ Cell Subsets in Patients with Early (EOA) and Late (LOA) Hip Osteoarthritis

**Figure 3:**
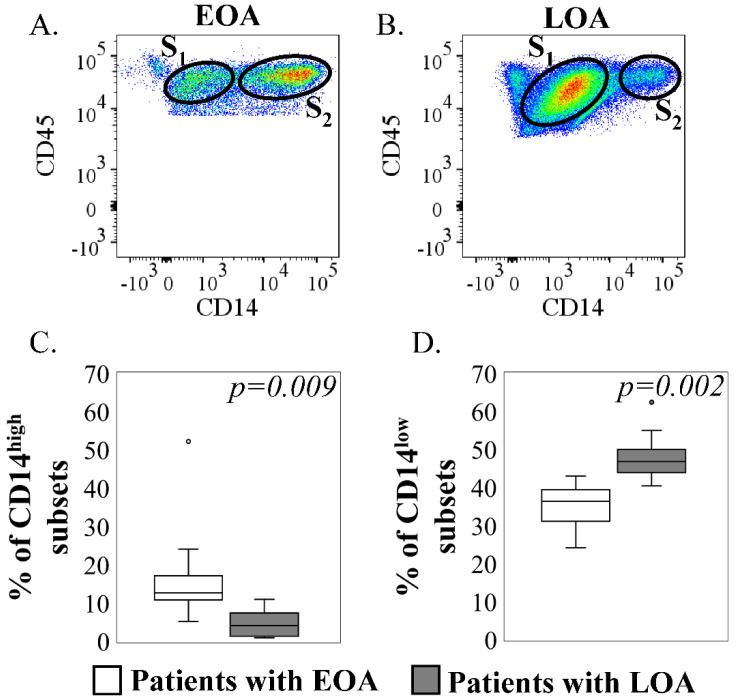
Differences in the proportion of CD14+ cell subsets in synovium from patients with early hip osteoarthritis (EOA) and late hip osteoarthritis (LOA) using flow cytometric analysis. Dot−plot analysis of CD14+ cells among synovial cells after CD45 gating in patients with EOA (**A**) and LOA (**B**). *X* axis, CD14; *Y* axis, CD45. S_1_ and S_2_ represent the CD14^low^ and CD14^high^ cell subsets, respectively. (**C**) The comparison of percentage of CD14^high^ subsets in CD45+ cells between EOA (white) and LOA (gray). (**D**) The comparison of percentages of CD14^low^ subsets in CD45+ cells between EOA (white) and LOA (gray). 3C represents the percentage of CD14^high^, and 3D represents the percentage of CD14^low^ cells of patients with EOA (white) and LOA (gray). Boxes represent median and IQR (interquartile range, 25−75th percentiles); small circle represent values outside 1.5 × IQR. Mann–Whitney *U*-test was used for analysis. *p* < 0.05 indicates statistical significance.

The eight tissue samples from EOA patients (one male, seven females) aged 43.0 ± 9.1 years and the eight tissue samples from LOA patients (two males, six females) aged 66.4 ± 10.0 years were analyzed using flow cytometry to investigate the differences in the proportion of CD14+ cell subsets. Figure 3A shows the dot plots of flow cytometric analyses in patients with EOA and LOA, depicting the subsets of CD14^low^ and CD14^high^ cells in synovium from both groups. The percentage of CD14^high^ subsets in CD45+ cells was significantly higher in hip early OA than in hip late OA (early OA, 17.6 ± 14.1%; late OA, 4.7 ± 3.5%; *p* = 0.009; Figure 3C). The percentage of CD14^low^ subsets in CD45+ cells was significantly lower in the early hip OA group (early hip OA, 35.0 ± 6.5%; LOA, 48.3 ± 6.7%; *p* = 0.002; Figure 3D).

## 3. Discussion

In this study, we found that synovial *CD14* expression in EOA was higher than that in LOA. In the flow cytometric analysis, there were heterogeneous subsets of CD14^low^ and CD14^high^ cells in the synovium from both EOA and LOA samples. The percentage of CD14^high^ subsets was higher in EOA than in LOA. These results suggest that CD14+ monocytes and macrophages may play an important role in the progression of degenerative changes in the synovium of EOA.

Several studies have indicated the characteristic patterns of macrophage expression between early OA and late OA in the knee joint. Benito et al. reported an immune histological analysis of an age-unmatched cohort in which synovial tissue from 10 patients with early knee OA demonstrated significantly more infiltrating CD68+ macrophages than synovial tissue from 15 patients with late knee OA [11]. Ostojic et al. reported immunofluorescence analyses that showed that the total number of Nuclear Factor Kappa-B (NF-kB), matrix metalloproteinase-9 (MMP-9), and inducible NO synthase (iNOS) on CD68+ macrophages, which play a distinctive role in the degradation of the cartilage, was increased in the synovial intima of early knee OA [25]. We previously reported that CD14+ cells derived from knee osteoarthritic synovium expressed TNF-α, which stimulated the cartilage degradation enzymes MMP-3 and ADAMTS4 [22,26]. In this study, we found that synovial *CD14* expression and the percentage of CD14^high^ cells in EOA was higher than that in LOA, suggesting that increased CD14+ monocytes/macrophages in the synovium may influence the progression of hip OA.

A previous study suggested that there were associations between CD14+ monocytes/macrophages and pain symptoms in patients with knee OA [23]. Daghestani et al. showed that soluble CD14 concentration in synovial fluids was associated with self-reported pain levels in knee OA (KL grades 1–4) [15]. In our study, CD14 expression was associated with pain in EOA before but not after matching. Aging can affect pain perception. Indeed, aging appears to be associated with increased pain thresholds, reflecting decreased pain sensitivity [27,28,29]. Therefore, the relationship between CD14 expression and pain score in an age-unmatched cohort may reflect the characterization of EOA in a younger population. In contrast, we found no significant difference in *CD14* expression in LOA patients with severe and moderate pain both before and after propensity matching. No correlations have been found between VAS rest and gait pain scores in LOA as defined by KL3 and 4 in our previous study [13]. The contribution of CD14-expressing cells in pain may be limited in both early and LOA. The sample size was small and may have limited statistical analysis. These small numbers may have resulted in insufficient power to detect a difference between groups after matching. To compare the association of CD14 expression with pain between early and late OA, further propensity-matched studies may be needed.

There were several limitations in this study. We did not conduct comparisons with healthy tissue and/or patients without pain. The number of synovial samples obtained from EOA during AS was limited, making the performance of RT-PCR and flow cytometry using the same samples difficult. We showed that there were different proportions of CD14^high^ and CD14^low^ subsets in EOA and LOA synovium, but we could not prove whether these subsets are directly involved in OA progression. Monocyte involvement in inflammation is based on CD14 and CD16 subsets, depending on their expression [30,31,32]. Further characterization of immunophenotypes is needed. It was difficult to distinguish between CD14^high^ and CD14^low^ cells using immunohistochemical procedures; the localization of these cells in the synovium remains unclear. Recently, methods such as the thermal analysis of synovial fluid, which are lower in cost, were developed to diagnose EOA and LOA [33]. Further investigation regarding the actual cost and accuracy will be necessary. Despite these limitations, our study provides valuable information about the association between the CD14+ cells in the synovium and progression of OA in patients with EOA.

## 4. Materials and Methods

### 4.1. Patients

Synovial tissue samples collected from a total of 130 consecutive patients at our center during surgery to treat hip OA were included. There were 42 patients who underwent hip AS and 88 patients who underwent total hip arthroplasty (THA). Exclusion criteria were previous surgery on the hip; current use or history of immunosuppressive medication; and hip OA secondary to pigmented villonodular synovitis, rheumatoid arthritis, trauma, idiopathic osteonecrosis of the femoral head, or rapidly destructive coxarthropathy. Samples were extracted from hyperemic synovial tissue lining the anterior joint capsule during both AS and THA procedures. The expression of synovial *CD14* mRNA was investigated by RT-PCR analysis in 34 and 80 patients who underwent hip AS and THA, respectively. Tissues from the remaining two sets of 8 patients each were used for flow cytometric analysis to evaluate the subsets in CD14+ cells.

### 4.2. Clinical Assessment

Radiographic assessment of hip OA progression was graded according to the Tönnis classification system: grade 0, no changes; grade 1, mild narrowing of the joint space, mild lipping at the joint margin, and mild sclerosis of the femoral head or acetabulum; grade 2, appearance of small bony cysts, additional narrowing of the joint space, and modest loss of femoral head sphericity; and grade 3, presence of large cysts, marked narrowing of the joint space, marked femoral head deformity [34]. To perform a more detailed radiographic assessment of Tönnis grades 0 and 1, we evaluated the presence of FAI cam morphology (α-angle ≥ 60°) and DDH (LCE angle ≤ 25°), which are associated with the onset of hip OA, on each radiograph [35,36]. All patients who underwent AS were classified as grade 0 or 1, and all patients who underwent THA were classified as grade 2 or 3. We divided the patients into grade 0 and 1 into an EOA group and those with grade 2 and 3 into an LOA group. Furthermore, in all patients who underwent hip AS, ALTs were observed intraoperatively and recorded.

Preoperative pain intensity was assessed using a visual analog scale for pain (VAS pain: 0 = no pain, 10 = worst possible pain). Patients with EOA and LOA were separated into severe (VAS pain ≥ 5) and moderate pain (VAS pain < 5) groups based on previous studies [37,38]. Clinical assessments were performed preoperatively at our outpatient clinic 1 month before each surgery. Table 1 compares patient background factors and clinical evaluations in patients with EOA and LOA who underwent RT-PCR analysis.

### 4.3. RT-PCR Analysis

Synovial samples were subjected to RNA extraction using a solvent (TRIzol, Invitrogen, Carlsbad, CA, USA) following the manufacturer’s protocol. First-strand cDNA was synthesized using a kit (superscript III RT™, Invitrogen), and used for RT-PCR analysis using another kit (SYBR™ Green, Qiagen, Valencia, CA, USA). RT-PCR was used to determine the *CD14* expression in synovial samples from EOA and LOA patients. Sequences of the PCR primer pairs used are provided in Table 2. *CD14* expression was normalized to that of glyceraldehyde-3-phosphate dehydrogenase. We evaluated the differences in *CD14* expression by EOA and LOA and by pain levels in EOA and LOA.

### 4.4. Flow Cytometric Analysis

Flow cytometric analysis was used to identify synovial CD14+ cell subsets from patients with EOA and LOA. Each synovial sample was digested with 2 mg/mL type I collagenase for 24 h at 37 °C. Cell fractions from each sample were reacted with R-phycoerythrin-conjugated anti-human CD14 (BioLegend, San Diego, CA, USA) and fluorescein isothiocyanate-conjugated anti-human CD45 (BioLegend). After washing twice in 2% fetal bovine serum, each fraction underwent flow cytometry and analysis according to the method of Ohashi et al. [39]. Each fraction was analyzed by flow cytometry (FACSVerse^TM^; BD Biosciences, San Jose, CA, USA). The data were analyzed using FlowJo, version 10.8.0 (FlowJo, Ashland, OR, USA). We previously found CD14^high^ and CD14^low^ subsets in synovium from advanced and end-stage hip OA [40]. Therefore, we compared the percentage of CD14^high^ and CD14^low^ subsets in early and late hip osteoarthritis patients.

### 4.5. Statistical Analysis

Results are expressed as the mean ± standard deviation, unless otherwise indicated. Categorical and continuous variables between two groups were compared using Pearson’s chi-squared test and the Mann–Whitney *U*-test, respectively. We used the non-parametric Mann–Whitney *U*-test as the continuous variables in all analyses which did not follow the normal distribution examined by the Shapiro–Wilk test. To create a matched cohort of EOA and LOA patients, a propensity score was calculated for each individual based on demographic characteristics (age, sex, height, weight, and body mass index). All statistical comparisons were conducted using commercial software (SPSS version 26.0, IBM, Armonk NY, USA). *p* values < 0.05 were considered statistically significant.

## 5. Conclusions

This comparative study between early and late OA using a propensity score-matched cohort showed that the synovial *CD14* expression and CD14^high^ subsets in EOA were higher than those in LOA. Increased *CD14* expression and the proportion of CD14^high^ subsets may be important features associated with EOA pathology. However, the contribution of CD14-expressing cells to pain may be limited in both early and LOA.

## Figures and Tables

**Table 1:** Clinical characteristics in early and late hip osteoarthritis patients before and after propensity matched score analysis.

	Before Propensity Matching			After Propensity Matching		
	EOA (N = 34)	LOA (N = 80)	*p*	EOA (N = 16)	LOA (N = 16)	*p*
Sex, female/male, N	25/9	69/11	0.102	12/4	12/4	1.000
Age, years	43.0 ± 16.0	65.9 ± 11.2	<0.001	53.9 ± 8.3	54.0 ± 9.1	0.956
Height, cm	164.4 ± 8.7	154.3 ± 8.6	<0.001	160.7 ± 10.8	162.7 ± 6.9	0.642
Weight, kg	60.6 ± 9.7	58.3 ± 13.7	0.221	61.3 ± 8.4	62.6 ± 15.9	0.867
BMI, kg/m^2^	22.7 ± 4.1	24.5 ± 4.5	0.052	23.8 ± 2.8	23.6 ± 5.6	0.590
Tönnis grade (0/1/2/3), N	27/7/0/0	0/0/21/59	<0.001	11/5/0/0	0/0/3/13	<0.001
α-angle ≥ 60°, N (%)	11 (32.4)			2 (12.5)		
LCE angle ≤ 25°, N (%)	14 (41.2)			10 (62.5)		
Combined, N (%)	5 (14.7)			2 (12.5)		
VAS pain ≥5/<5 cm, N	20/14	53/27	0.450	12/4	12/4	1.000

The patients with Tönnis classification grade 0 or 1 constituted the EOA group, and the patients with Tönnis classification grade 2 or 3 constituted the LOA group. Data are reported as mean ± standard deviation. Continuous variables were calculated using the Mann–Whitney *U*-test and categorical variables were calculated using the chi-squared test. Statistically significant *p* values (<0.05) are in boldface. Abbreviations: EOA, early hip osteoarthritis; LOA, late hip osteoarthritis; BMI, body mass index; LCE, lateral-center edge; Combined, α-angle ≥ 60° and LCE angle ≤ 25°; VAS, visual analog scale.

**Table 2:** Sequences of the primers.

Primer	Sequence (5′-3′)	Product Size (bp)
CD14-F	TCCCTCAATCTGTCGTTCGC	150
CD14-R	ATTCCCGTCCAGTGTCAGGT	
GAPDH-F	TGTTGCCATCAATGACCCCTT	202
GAPDH-R	CTCCACGACGTACTCAGCG	

Abbreviations: GAPDH, glyceraldehyde-3-phosphate dehydrogenase; bp, base pairs.

## Data Availability

The data presented in this study are available on request from the corresponding author.
